# Cough Mixture

**Published:** 1879-09

**Authors:** 


COUGH MIXTURE.—J. Milner Fothergill says hydrobomic acid with spirit of chloroform and syrup of squill—and if the case be that of a very agreeable lady, and a favorite patient, a few drops of spirit of nutmeg be added—constitutes an excellent and palatable cough medicine.—*Western Lancet.*

---

SEALING WAX FOR FRUIT CANS.—

Melt together:

| | | |
|---|---|---|
| Yellow wax | 1 | ounce. |
| American vermillion | 3 | ounces. |
| Gum shellac | 5 | ounces. |
| Rosin | 16 | ounces. |

Run into moulds.

---

DR. J. H. LOGAN, Professor of Chemistry in the Atlanta Medical College, has in press a hand-book of the Elements of Chemistry, which is specially adapted to the requirements of students. The work will be completed by next month, and we are satisfied that it will be found indispensable to all beginners in the study of chemistry.

